# Early hyaluronidase use in preventing skin necrosis after treatment with dermal fillers: Report of two cases

**DOI:** 10.12688/f1000research.15568.2

**Published:** 2019-04-03

**Authors:** Francesco Ciancio, Maria Stella Tarico, Giuseppe Giudice, Rosario Emanuele Perrotta

**Affiliations:** 1 Department of Plastic and Reconstructive Surgery, University of Bari, Bari, 70124, Italy; 2 Department of Plastic and Reconstructive Surgery, University of Catania, Catania, 95100, Italy

**Keywords:** dermal filler, skin necrosis, filler complications, vascular complications

## Abstract

Injection of dermal fillers, like hyaluronic acid (HA), is a safe procedure, with few and transient side effects such as erythema, bruising and swelling etc. The aim of this report is to provide our protocol for the early treatment of necrotic complications after facial treatment with dermal fillers.

We present two cases of skin suffering of the face after dermal infiltration of HA, treated successfully with our early protocol. Our protocol includes the early infiltration of hyaluronidase in the treated areas. We start with infiltration of hyaluronidase distributed over the area to be treated through micro-injections with dosage 40 IU per cm ^2^. Our protocol includes the use of systemic corticosteroids for 4 days, anti-aggregation therapy, oral antibiotic, topical cream with nitric oxide and compresses with gauze and warm water.

In the skin complications after dermal filler treatment, marked pain and characteristic reticulated erythema in the skin distribution of the affected vessels is often developed. Due to the implementation of our protocol in these patients, we managed to avoid an irreversible necrotic complication of the face in both cases.

In this report, our protocol was compared with results published in the literature and allowed us to avoid complications such as skin necrosis with permanent damage.

## Introduction

The use of dermal fillers is increasingly required in cosmetic surgery ^1^. Injection of dermal fillers is a safe procedure, with few and transient side effects. However, cases of skin necrosis have been reported, including with involvement of vision and ocular globe ^2^. The cause is an impediment of the blood supply by compression and/or obstruction of the vessel(s) with filler material, and/ or direct injury to the vessel ^3, 4^. Several therapeutic approaches have been described ^5– 7^.

The aim of this report is to present our protocol for the early treatment of vascular complications after facial rejuvenation with dermal fillers in order to avoid skin necrosis. We present two cases of vessel damage and skin suffering of the face after dermal infiltration of hyaluronic acid (HA), which occurred in 2017 and was treated successfully with our protocol.

## Protocol

Infiltration of hyaluronidase is performed firstly, at a deep dermal level, and is distributed over the area to be treated through micro-injections at a dosage of 40 IU per cm ^2^. The distribution is homogeneous except for nodular areas in which a double dose is infiltrated (in any case, at least 150 IU of hyaluronidase in 1 ^th^ infiltration is used). The lithic enzyme must be infiltrated at the dermal level, homogeneously distributed, in order that from the point of infiltration it is distributed to the vessels by diffusion. The rational use of hyaluronidases is to breakdown HA particles and allow reabsorption. It is well known that hyaluronidases are inactivated by the immune system ^8^, so the treatment must be repeated to obtain an adequate concentration. In our clinical practice, we use the maximum dosage (40IU per cm ^2^) for three consecutive days. The areas to be treated are established on the basis of clinical signs of damage/ischemia distribution for which, in selected cases, maintenance doses (40 UI/cm ^2^) can be applied in the areas most affected. The treatment is repeated after a few hours if the ischemic area shows no improvement with double daily doses but, in all cases, never extended beyond 72h.

Systemic corticosteroids for four days (prednisone 25 mg/24h per os) to reduce edema and increase microcirculation perfusion, oral salicylic acid 100 mg per os like anti-platelet, antibiotic prophilaxis (levofloxacin 500mg / 24h for 4 days) for any infections, topical cream with nitric oxide (3 times a day) to improve blood perfusion, antibiotic therapy, topical cream with nitric oxide, and compresses with gauze and warm water are also recommended in this protocol. In the following days, the evolution of clinical signs should be monitored and therapy continued if needed.

The protocol must be implemented as soon as possible, specifically the cortisone drugs are used in the first 24 hours and continue until the fourth day, the treatment with acetylsalicylic acid 100 mg starts in the first 24 hours and lasts for 10 days, the antibiotic prophylaxis is established. in the first 24 h and continues for 4 days.

## Case 1

**Figure 1.**
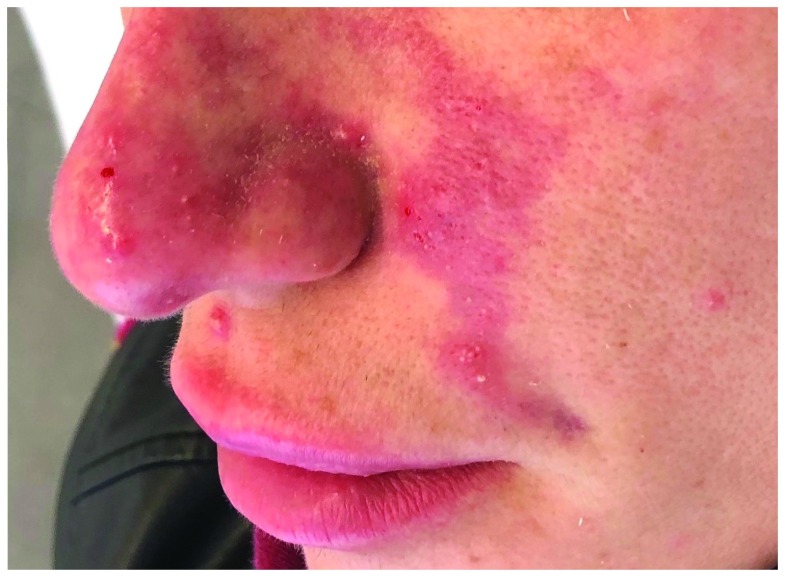
A 36-year-old female patient with skin suffering in the left naso-labial fold region after hyaluronic acid infiltration from dermal fillers. Three days after dermal filler treatment, erythematous and blister formation was observed along the nasolabial vessels. Part of the erythema extends to the middle of the nose.

In June 2017 a 36-year-old female patient was admitted for treatment of infiltration of HA-based dermal fillers. The patient had received treatments with dermal fillers in the past without adverse reactions. Immediately after the dermal filler procedure, the treated areas appeared in good condition without signs of skin suffering. Three days later, at a follow-up examination, the left treated area appeared cyanotic and swollen despite the patient not complaining of discomfort. The skin appeared erythematous with distribution along the left nasolabial folds up to the lateral nasal wall, and the capillary refill time appeared slow or absent ( Figure 1). Consequently, treatment with the protocol, as stated above, was performed immediately. We used 40UI of hyaluronidase per cm ^2^, two times a day for 3 days. The patient received acetylsalicylic acid 100 mg / 24h for 10 days, prednisone 25mg / 24h for 4 days, levofloxacin 500mg / 24h for 4 days, topical cream with nitric oxide 2 times a day and compresses with gauze and warm 3 times a day.

**Figure 2.**
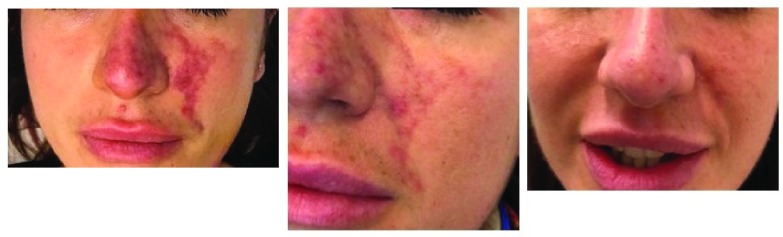
A 36-year-old female patient with hyaluronic acid infiltration from dermal fillers treated with the early protocol. Left panel: 7 days after first treatment; middle panel: after 15 days; right panel: clinical check after 45 days. It should be noted that there is no scarring in the final image.

Necrotic complications of the face were avoided in this patient ( Figure 2).

## Case 2

**Figure 3.**
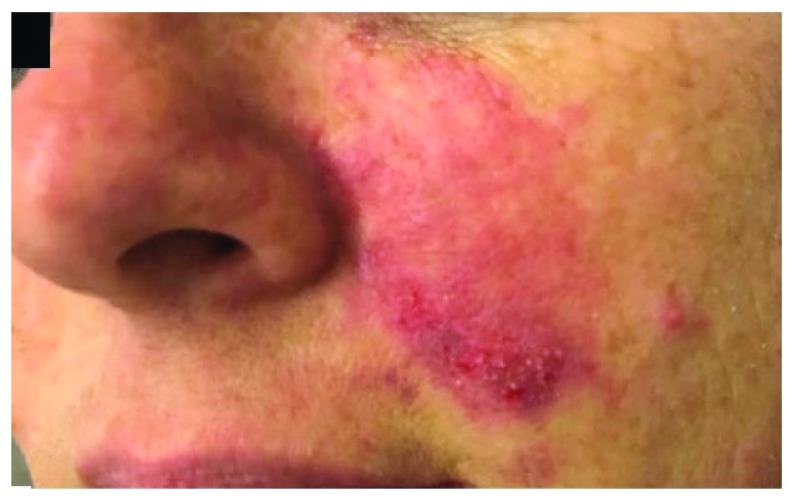
A 45-year-old woman with skin suffering in left naso-labial fold region after hyaluronic acid infiltration from dermal fillers. There is an erythematous halo, blisters and livedo reticularis in the middle third of the left cheek.

In August 2017 a 45-year-old woman was treated with HA to fill the region of nasolabial folds. In the past the patient had received similar treatments without adverse reactions. At the clinical check after three days, the patient shows signs of skin suffering. Compared to Case 1 the erythematous area was smaller with distribution retained to the medial region of the cheek ( Figure 3). Treatment with the protocol, as stated above, was performed immediately and 40UI/cm ^2^ of hyaluronidase was injected every 12 h per 2 days, after only 1 dose for the third day. Systemic corticosteroids, antiplatelet therapy, antibiotic therapy and local topics were used according to protocol, as expressed in Case 1.

**Figure 4.**
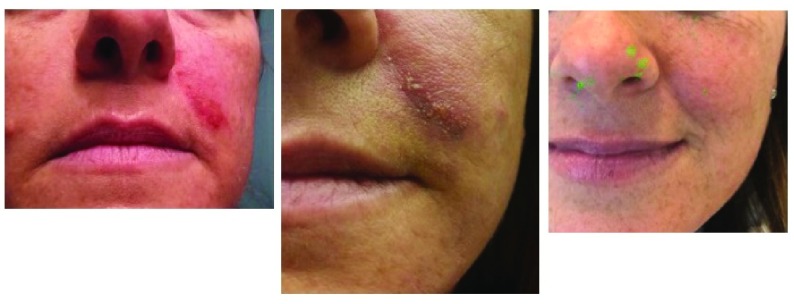
A 45-year-old female patient with hyaluronic acid infiltration from dermal fillers treated with the early protocol. Left panel: the erythematous lesion has decreased in intensity after 7 days from treatment; middle panel: after 12 days; right panel: after 45 days. The green dots in the picture are the result of a damaged camera, we have not modified the image.

Necrotic complications of the face were avoided in this patient ( Figure 4).

## Discussion

Damage after dermal fillers can lead to severe consequences, such as skin necrosis, and the involvement of ocular, nerve and muscle structures ^2^. Skin necrosis is the most significant complications after dermal filler. The incidence of vascular damage after use of fillers has been estimated at 3–9 per 10,000 for HA products, but the true incidence of this complication is unknown ^9, 10^. The skin necrosis after dermal fillers injection is an emergency, the best treatment is often the quickest. The gold standard is prompt injection of hyaluronidase with a dose of 40 IU per cm2 of affected area. Some authors have described oral antiplatelet drugs, like cardioaspirin while some have cited the use of vasodilator drugs. Today again there is no international standard protocol for the treatment of these complications. In our cases, the patients manifested skin problems probably from filler emboli or direct destruction of the vessels during needle manoeuvres. Interestingly our patients did not experience any pain or discomfort during and after the HA dermal filler procedure. In the literature, some manoeuvres have been reported to reduce the risk of vascular damage, such as aspiration during infiltration ^11^, low pressure injection, continuously moving the needle or cannula while injecting, injection of small quantities (maximum 0.1 mL of filler per pass) ^12^, observing skin changes during the immediate post-injection phase, and excellent knowledge of anatomy ^13– 15^. Typically in areas with terminal vascular circulation, the cutaneous vessels suffering may be more likely; in our cases it is one of the most vascularized areas of the face, the nasolabial folds ^15^.

In this kind of complication, an early treatment is the best choice. The gold standard is prompt injection of hyaluronidase ^16^.

De Lorenzi presents a protocol with high doses of hyaluronidase ^8^, where the dosage of hyaluronidases is quantified on the basis of the regions of the face affected (for example: for the glabellar region 500 IU of hyaluronidase). We agree with the De Lorenzi that the use of elevated quantities of hyaluronides is required for the treatment of adverse events of vascular filler; however, we believe that a distribution per cm ^2^ is more precise. More treatments has been described but there is no international standard protocol for the treatment of these complications.

As expressed by various Societies of Plastic and Aesthetic Surgery, to minimize the incidence of this type of damage it is essential to contact qualified and trained medical personnel, who follow modern international protocols ^17– 19^.

The strengths of our protocol is certainly the result with no residual mark of skin suffering that has been seen in our cases, while the major limitation is the necessary execution of the protocol within 72h from the damage.

## Conclusion

Today, the best solution for vascular damage is to categorically avoid dermal fillers treatments with non-medical and untrained personnel. This report aims to offer present a protocol for the early treatment of vascular damage after dermal fillers. Our early-implementation protocol has been compared with results presented in the literature and allowed us to avoid complications such as skin necrosis with permanent damage ^20, 21^.

## Consent

Written informed consent was obtained from the patients for the publication of this case report and any associated images.

## Data availability

All data underlying the results are available as part of the article and no additional source data are required.
