# Supplementary material for: Early hyaluronidase use in preventing skin necrosis after treatment with dermal fillers: Report of two cases
Source: F1000Res. 2019 Apr 3;7:1388. Originally published 2018 Sep 3. [Version 2] doi: 10.12688/f1000research.15568.2 (PMC6449787; doi:10.12688/f1000research.15568.2)
Supplement: Peer review history

# Peer review: Reviewer response for version 2

**DOI:** 10.5256/f1000research.20633.r46715

**Published:** 2019-04-10

**Authors:** Gottfried Lemperle

**Affiliations:** 1 Division of Plastic Surgery, San Diego School of Medicine, University of California, San Diego, CA, USA

My impression is still that both cases with superficial necroses would have healed easily by nature, only- and that Dilorenzi´s experiments on hyaluronidase penetration through dead arteries are not sufficient proof to recommend intralesional hyaluronidase injections: there is no hyaluronic acid outside of the arteries.

I have read this submission. I believe that I have an appropriate level of expertise to state that I do not consider it to be of an acceptable scientific standard, for reasons outlined above.

# Peer review: Reviewer response for version 2

**DOI:** 10.5256/f1000research.20633.r46716

**Published:** 2019-04-03

**Authors:** Robert J. Besaw

**Affiliations:** 1 Beth Israel Deaconess Medical Center, Boston, MA, USA

Ciancio and colleagues have provided a revised version of their article which includes additional information on rationale, dosing, and administration of the components of their newly proposed protocol. With this information, the protocol should be much more reproducible for others in the community. It will be up to the community to further determine clinical benefit for this regimen in larger studies.

I have read this submission. I believe that I have an appropriate level of expertise to confirm that it is of an acceptable scientific standard.

# Peer review: Reviewer response for version 1

**DOI:** 10.5256/f1000research.16981.r45979

**Published:** 2019-04-01

**Authors:** Robert J. Besaw

**Affiliations:** 1 Beth Israel Deaconess Medical Center, Boston, MA, USA

Ciancio and colleagues present a new protocol for preventing skin necrosis for patients undergoing treatment of dermal fillers. As the use of dermal fillers become increasingly common, this could offer a new approach to mitigating potential serious adverse events.

 This study offers an interesting new proof of concept with two cases that underwent the protocol. Ideally, the authors will next present a larger observational study to further support their concept and demonstrate the benefits of this regimen. 

 To make sure that others would be able to duplicate such a procedure, it's important to be specific. The authors should take the time to explain in more detail why each of the components is included in the regimen, i.e. what each piece is adding. Also, would recommend detailing when systemic corticosteroids should begin (prior to rest of the regimen? after?). If doses/timing is all done on a case-by-case basis, then this must be stated. 

 Overall, nice outcomes in two cases from an interesting protocol. Will require a larger study to demonstrate if this protocol warrants more widespread uptake.

I have read this submission. I believe that I have an appropriate level of expertise to confirm that it is of an acceptable scientific standard, however I have significant reservations, as outlined above.

# Response

**Published:** 2019-04-01

**Authors:** Francesco Ciancio

**Affiliations:** University of Bari, Italy

Revision:

As recommended by the Review we added (in red) details of the dosage on the presented protocol, we have included the rationale of use for each treatment. We have added a paragraph in the section of the Protocol indicating the times of use of the drugs and when to begin treatment with each category of drugs.

# Peer review: Reviewer response for version 1

**DOI:** 10.5256/f1000research.16981.r41307

**Published:** 2018-12-04

**Authors:** Gottfried Lemperle

**Affiliations:** 1 Division of Plastic Surgery, San Diego School of Medicine, University of California, San Diego, CA, USA

Sorry to say: but these 2 cases are not representative for an outcome of any therapy; they would have been solved by themselves, i.e. by Nature within one week. Check your own publication (below) or google images: much worse superficial necroses and infections healed without obvious scarring. - My PC contains at least 10 serious cases from Brazil and elsewhere, where cosmeticians and even hairdressers are allowed to inject dermal fillers.

 The reason for skin necrosis in the face is always intra-arterial injection: therefore, its prevention is using a cannula or better: moving the needle or a cannula back and forth during injection. The facial artery can easily be palpated from intraorally just above the mucosa and way beneath the nasolabial fold. To inject HA soo deeply shows the lack of knowledge of the anatomical layers of the face.

 Hyaluronidase is a logical therapy (and the present Gold Standard, I know) - if it could be injected into the embolized artery. DeLorenzi´s experiments on blocked dead arteries in hyaluronidase are not convincing that this enzyme dissipates through the arterial wall from the outside. I just reviewed a manuscript from Beijing where similar experiments in a living rabbit ear showed slight recovery of the necrosis only when hyaluronidase was injected intra-arterially within the first 4 hours.

 It is difficult to demonstrate an effect of any therapy in emergencies.

 To make it short, as long as there is no proof that hyaluronidase enters blocked arteries from the outside (why should it?) we should stick to an immediate physical procedure to try to spread the intra-arterial HA by retrograde scratching with a fingernail through the oral mucosa and emptying it thereby - or massive massaging of the blocked area in all directions hoping to distribute the HA to other branches. I have not done it - but it sounds logical to me.

I have read this submission. I believe that I have an appropriate level of expertise to state that I do not consider it to be of an acceptable scientific standard, for reasons outlined above.

## References

[1] DeLorenziC: Transarterial degradation of hyaluronic acid filler by hyaluronidase. *Dermatol Surg*.2014;40(8) : 10.1097/DSS.0000000000000062 832-41 10.1097/DSS.0000000000000062 25022707

[2] DeLorenziC: New High Dose Pulsed Hyaluronidase Protocol for Hyaluronic Acid Filler Vascular Adverse Events. *Aesthet Surg J*.2017;37(7) : 10.1093/asj/sjw251 814-825 10.1093/asj/sjw251 28333326

[3] ZhuGZSunZSLiaoWXCaiBChenCLZhengHHZengLLuoSK: Efficacy of Retrobulbar Hyaluronidase Injection for Vision Loss Resulting from Hyaluronic Acid Filler Embolization. *Aesthet Surg J*.2017;38(1) : 10.1093/asj/sjw216 12-22 10.1093/asj/sjw216 28333176

[4] ChenYCWuHMChenSJLeeHJLirngJFLinCJChangFCLuoCBGuoWY: Intra-Arterial Thrombolytic Therapy Is Not a Therapeutic Option for Filler-Related Central Retinal Artery Occlusion. *Facial Plast Surg*.2018;34(3) : 10.1055/s-0037-1621730 325-329 10.1055/s-0037-1621730 29378380

[5] KimHJKwonSBWhangKULeeJSParkYLLeeSY: The duration of hyaluronidase and optimal timing of hyaluronic acid (HA) filler reinjection after hyaluronidase injection. *J Cosmet Laser Ther*.2018;20(1) : 10.1080/14764172.2017.1293825 52-57 10.1080/14764172.2017.1293825 29199877

# Response

**Published:** 2018-12-15

**Authors:** Francesco Ciancio

**Affiliations:** University of Bari, Italy

It is necessary to clarify about the review of Gottfried Lemperle. 

We agree on the use of blunt cannulas and on infiltration techniques with continuous moviments, small quantities of filler and anything else procedure to reduce the risks of ischemic damage from filler infiltration.

The reviewer affirms that “these 2 cases are not representative for an outcome of any therapy; they would have been solved by themselves, i.e. by Nature within one week” we do not agree with him.

The signs of vascular complications after dermal fillers are described in the literature ^1^. In fact, the early signs are blisters (3 days), erithema, livedo reticularis (up to a few days) and then crusting, necrosis (after days 6), slough, and finally healing by secondary intention − a process that may take six weeks or more. Therefore, in our clinical opinion, the photos shown in the paper are indicative of early signs of vascular compromise from dermal fillers and require integrated early treatment. The presumption that the scar results would be the same even without treatment must be scientifically proven.

We do not agree when he says "Delorenzi's Experiments on Online Blonde Dead Arteries in Hyaluronidase Are Are not Convincing This Enzyme Dissipates Through The Arterial Wall From The Outside ... to Make It Short, As Long As there is not Proof That Hyaluronidase Enters Blocked Arteries from the Outside (Why Whyu Shted it?)" ^2^.

In literature there are many papers that state the effectiveness of the use od hyaluronidases in the subcutaneous tissue, near the affected area. The skin damage may result from extrinsic obstruction of the vessels so the necrosis may also occur secondary to local edema or to occlusion of adjacent vessels, secondary to the hydrophilic properties of the product, motivated the extravascular use of hyaluronidases.  Use of this enzyme around the vessels is finalized to reduce the extrinsic vessel’s obstruction by hyaluronic acid. Several studies showed that the injection of hyaluronidase, throughout the area around the vascular occlusion point, promote its intravascular penetration and facilitate removal of the HA that is obstructing the vessel ^3-4-5^. De Lorenzi's experiment on sections of arteries and facial veins appears interesting and the concept of "flooding" the affected area deserves respect. Until there is no contrary evidence, it is a logical and appropriate rational. So we are in disagree with the reviewer. 

1. Urdiales-Gálvez F, Delgado NE, Figueiredo V, et al.: Treatment of Soft Tissue Filler Complications: Expert Consensus Recommendations. Aesthetic Plast Surg. 2018; 42(2): 498–510.  
2. DeLorenzi C: Transarterial degradation of hyaluronic acid filler by hyaluronidase.Dermatol Surg. 2014; 40 (8): 832-41 
3. DeLorenzi C: New High Dose Pulsed Hyaluronidase Protocol for Hyaluronic Acid Filler Vascular Adverse Events.Aesthet Surg J. 2017; 37 (7): 814-825 
4. Hirsch RJ, Cohen JL, Carruthers JD (2007) Successful manage- ment of an unusual presentation of impending necrosis following a hyaluronic acid injection embolus and a proposed algorithm for management with hyaluronidase. Dermatol Surg 33(3):357–360 
5. Bachmann F, Erdmann R, Hartmann V, Wiest L, Rzany B (2009) The spectrum of adverse reactions after treatment with injectable fillers in the glabellar region: results from the Injectable Filler Safety Study.  Dermatol  Surg 35(Suppl 2):1629–1634 

# Peer review: Reviewer response for version 1

**DOI:** 10.5256/f1000research.16981.r38457

**Published:** 2018-10-03

**Authors:** Nicolò Bertozzi

**Affiliations:** 1 Department of Medicine and Surgery, Division of Plastic Surgery, Cutaneous, Mininvasive, Regenerative and Plastic Surgery Unit, University Hospital of Parma, University of Parma, Parma, Italy

Very good written article about the author's experience with the use of hyaluronidase in preventing skin necrosis after dermal filler infiltration. Their protocol is clearly stated and the pictures are very representative of the clinical course of the two patients treated. Discussion could be more complete by citing further authors. Thanks for your effort.

I have read this submission. I believe that I have an appropriate level of expertise to confirm that it is of an acceptable scientific standard.
